# Genetic evaluation of different graded Holstein Friesian × Local (HF × L) crossbred breeding bulls of Bangladesh

**DOI:** 10.5455/javar.2025.l901

**Published:** 2025-04-16

**Authors:** Md. Forhad Ahmed Hridoy, Sadia Afrin Siddiqua, Doo Ho Lee, Yeong Kuk Kim, Md. Shane Khoda, Monira Akter Mou, Abul Kashem Fazlul Haque Bhuiyan, Seung Hwan Lee, Mohammad Shamsul Alam Bhuiyan

**Affiliations:** 1 Department of Animal Breeding and Genetics, Faculty of Animal Husbandry, Bangladesh Agricultural University, Mymensingh, Bangladesh; 2 Division of Animal and Dairy Science, Chungnam National University, Daejeon, Republic of Korea; 3 Quantomic, Daejeon, Republic of Korea; 4 Senior Scientific Officer, Central Artificial Insemination Laboratory, Dhaka, Bangladesh

**Keywords:** Breeding Value, Bangladesh, Crossbred Bull, Genotype, Genetic Parameters, Selection

## Abstract

**Objective:**

The aim of this study was to estimate genetic parameters, breeding value, and ranking of Holstein Friesian × Local (HF × L) crossbred sires based on multi-trait selection index information.

**Methods:**

A total of 51 HF × L crossbred breeding bulls of three different genetic groups (50%HF × 50%L, 62.5%HF × 37.5%L, and 75%HF × 25%L) managed at Central Cattle Breeding and Dairy Farm (CCBDF) were evaluated based on 4,319 half-sib progeny performance data. The descriptive statistical analysis was performed using the R package. Genetic parameters were estimated using BLUPF90 by a single-trait animal model. A selection index was constructed using adjusted breeding values multiplied by variable economic weightage for each trait.

**Results:**

Genotype had significant effects on the investigated semen quality attributes (*p <* 0.05), where 75%HF × 25%L and 62.5%HF × 37.5%L crossbred genotypes both differed significantly from 50%HF × 50%L crossbreds (*p <* 0.05). In general, better productive and reproductive performances were found with the progression of HF inheritance. Daughters of 75%HF × 25%L crossbred bulls showed the highest average performance in birth weight (27.20 ± 0.09 kg), daily milk yield (8.55 ± 0.06 l), peak milk yield (10.44 ± 0.07 l), and lactation length (233.53 ± 0.85 days).

The study investigated variance components of eight productive and reproductive traits such as the birth weight of calf, age at first conception, service per conception, daily milk yield, peak milk yield, lactation length, and calving interval where estimated heritability ranged from 0.09 to 0.32. Genetic correlations among the considered traits were found to be mostly weak. Among the top 20%, breeding bulls belonged to 75%HF × 25%L and 62.5%HF × 37.5%L crossbred groups.

**Conclusion:**

This study provides insightful information on the genetic evaluation of different graded bulls that could be the basis for the proven crossbred breeding bulls’ selection process at CCBDF.

## Introduction

Cattle is one of the important components of the integrated farming system of Bangladesh with diversified roles in the mitigation of malnutrition, unemployment, and women empowerment, as well as increasing the fertility of agricultural land and earning foreign currency. In Bangladesh, about 62% of livestock producers raise cattle as their main animal [1]. The total number of cattle is about 24.55 million, contributing a 1.47% share of the gross domestic product [2]. The available cattle genetic resources of Bangladesh are mainly classified as non-descript Deshi, Pabna cattle, Red Chittagong cattle, Munshiganj cattle, North Bengal Grey, Crossbred cattle with various degrees of exotic inheritance, and exotic temperate and tropical breeds such as Holstein Friesian (HF), Sahiwal (SL), and Jersey [3,4]. Due to the lower productivity of the existing indigenous cattle of Bangladesh, crossbreeding or upgrading has been practiced to fulfill the country’s growing demand for milk and meat. A considerable number of crossbreds or upgraded animals are discernible nowadays across the country due to the resultant upgradation scheme operated by public-private breeding service providers. The most preferred HF × L and SL × L crossbreds produce a significant amount of milk in an economical way [5].

Selection and breeding are two important tools for improving the genetic potential of the existing genetic resources. Importantly, a potential bull can contribute to rapid herd development by transmitting half of the genetic potentials to its progenies, and the progeny performance is the true reflection of the sire’s genetic potentials [6]. In Bangladesh, the selection of sires, dams, and potential heifers has just been based on phenotypic performance data rather than the genetic merit of animals due to the absence of a functional animal identification and herdbook-based animal recording system [3]. The selection of genetically superior bulls based on semen quality and daughters’ performance, along with genomic information, could mitigate the existing limitations in Bangladesh. Heritability estimates and their use in the prediction of genetic merit are the key components of selection. Estimation of genetic parameters (heritability, genetic correlations, and phenotypic correlations) and breeding value is a prerequisite for genetic evaluations of animals and to ascertain the degree of genetic relationship or differences among the individuals of a population. Accurate estimates of genetic parameters are essential for implementing sound breeding programs and for accelerating the progress of ongoing programs [7].

On the other hand, the construction of a selection index involving multiple traits provides better estimates for selecting animals with more accuracy. The aggregated index value of an individual is directly related to economic weights given for the considered traits [8] and is largely dependent on the production system and purpose of animal rearing. The Central Cattle Breeding and Dairy Farm (CCBDF), under the Department of Livestock Services (DLS), plays a vital role as a pioneer for conducting artificial insemination (AI) programs in Bangladesh. The cattle upgradation program in Bangladesh has been operated for the last five decades, centering on this breeding farm. CCBDF disseminates frozen semen throughout the country in connection with 15,389 artificial insemination sub-centers/points [2]. Therefore, a precise selection of breeding bulls of CCBDF can contribute more to disseminating genetic potentiality to the progenies through semen. In this study, several crossbred sires of CCBDF that have been producing semen for the last 4–6 years and have progeny performance records at the farm level were included for genetic evaluation. The study aims to select top-ranked bulls through the establishment of a farmers’ participatory phenotypic performance database and genetic evaluation of bulls based on index values considering the economic weights of multiple traits. This will, in turn, assist the relevant personnel of DLS and farmers to decide on further breeding based on the evaluated information on breeding bulls of CCBDF.

## Materials and Methods

### Animal care

Institutional Animal Care and Use Committee approval is not needed for this study as there is no direct involvement of animals to generate objective data. All sorts of data on semen parameters were collected from the data sheet maintained at CCBDF for each animal, and the daughters’ productive and reproductive performance data were collected through the use of individual herdbooks.

### Breeding bulls and phenotypes

A total of 51 sires under three different genetic groups were investigated in this study. The genotypes of the breeding bulls were 50%HF × 50%L (08), 62.5%HF × 37.5%L (12), and 75%HF × 25%L (31). These bulls have been maintained at CCBDF, Savar, Dhaka, under DLS, Bangladesh. The age of the bulls ranged from 5 to 8 years, and they are still being used for semen production. Semen collection has been practiced twice per week, and information on four semen parameters, such as semen volume in ml, concentration in million/ml, mass motility in percentage, and post-thaw motility in percentage. Semen collection was performed using the artificial vagina method, and frozen semen samples were stored in liquid nitrogen for further use. The semen volume was measured directly from the graduated collection vial by eye estimation, and semen concentration was measured by photometer SDM6 (Minitab GmbH 12,500, Germany). Measurement of mass motility was done by putting one little drop of sperm on a clean pre-warmed (37°C) slide and inspecting it under a microscope, and sperm movement was evaluated by Andro Vision AXIO (version 6.0.1, Minitub GmbH 12,500, Germany). With the reading, the program parameters were adjusted; minimum motile speed (microns/sec): 28; maximum burst speed (microns/sec): 600; distance scale factor (microns/sec): 7.50; minimum cell size (pixels): 6; maximum cell size (pixels): 6; sperm count per field analysis >1,000; minimum number of fields per sample: 3; were included in the sire evaluation process. Frozen semen distribution has been performed countrywide according to the roadmap of DLS. Besides, feeding, vaccination, and management practices of breeding bulls were almost similar to the experimental animals. Both roughage and concentrate were provided based on their live weight.

### Establishment of progeny performance database

A total of 4319 daughters’ productive and reproductive performance data sired by 51 breeding bulls of CCBDF were collected from Dhaka, Khulna, Jessore, Rangpur, Bogura, and Chattogram districts of Bangladesh. A simple two-page herdbook was maintained for each animal to keep records of lifetime events. Animal selection and data collection were performed by the assigned personnel under the supervision of the District Artificial Insemination Center, DLS, and the Krishi Gobeshona Foundation funded project. The daughter’s performance database was established between the years 2019 and 2022. The studied productive and reproductive traits were birth weight of calf (BWT) in kilograms (measured within 24 h of birth), age at first conception (AFC) in months (the age when an animal produces mature, fertile ova, and conceives), service per conception (SPC) (the number of inseminations required per successful conception), daily milk yield (DMY) in liters per day (the average milk production per cow each day throughout the lactation period), peak milk yield (PMY) in liters per day (the highest milk production on a single test day during the lactation period), lactation length (LL) in days (the period from calving to dry-off), days open (DO) in days (the interval between parturition and the subsequent conception), and calving interval (CI) in months (the period between consecutive calving intervals). The management system was predominantly semi-intensive, with smallholder dairy farmers managing herds of two to five cows.

### Statistical analyses

The collected data was tested for normal distribution. Extreme values were excluded from the data sheets by adopting two standard deviation functions using Microsoft Excel 2019. Phenotypic performance data were analyzed by the Agricole package implemented by R software [9]. Mean separation was tested using the Pastecs package in R [10].

The generalized linear model (GLM) was used with the following model for the evaluation of sires’ data:

*Y_ikj_* = μ + *G_i_* + *P_J_* + *A_K_* + *e_ijk_*

where *Y_ikj_* is the vector of phenotypes; μ is the overall mean; *G_i_*is the effect of genotype; *P_J_* is the effect of production year; *A_K_* is the effect of age; and *e_ijk_* is the random residual error.

The daughters’ performance data of breeding bulls were analyzed by the GLM using the following statistical model:

*Y_ikjl_*=μ+*S_i_*+*P_J_*+*L_k_*+*LS_l_*+*e_ijkl_*

where, *Y_ikjl_* is the vector of phenotypes; μ is the overall mean; *S_i_* is the effect of sire; *P_J_* is the effect of parity; *L_k_* is the effect of location; *LS_l_* is the interaction effect between location and sire; and *e_ijkl_* is the residual error.

The variance components, heritability, genetic correlation, and breeding value were estimated using the BLUPF90 program [11], assuming that all effects in the models were independent and distributed normally. The following equation was used for heritability and genetic correlations estimation:

$h^{2}\;=\;\frac{\sigma_{A}^{2}}{\sigma_{P}^{2}}\;\mathrm{and}\;r_{g}\;\sigma \;\frac{\sigma_{\mathrm{aij}}}{\sqrt{\sigma_{\mathrm{ai}}^{2}\;\sigma_{\mathrm{aj}}^{2}}}$

where h^2^ is the estimated heritability; σ^2^_A_ is the additive genetic variance; σ^2^_P_ is the phenotypic variance; r_g_ is the genetic correlations; σ^2^_*ai*_ is the additive genetic variance for trait i; σ^2^_*aj*_ is the additive genetic variance for trait j; and σ_*aij*_ is the additive genetic covariance between traits i and j.

### Construction of selection index

A multi-trait selection index was constructed based on the aggregated genetic worth or breeding values of the considered traits. A selection index (total score) was constructed based on the sum of standardized breeding values multiplied by the relative economic weights of eight important productive and reproductive traits using variance-covariance matrices. For that, variance components and heritability were calculated to get the breeding value of the respective trait. Here, eight economically important traits were considered for the construction of the selection index. The economic weights of each trait were calculated as:

*b* = *P*^-1^*G*a

where G is a genotypic variance-covariance matrix for traits; P is an inverse of the phenotypic variance-covariance matrix of correlated indicator traits; a is a column vector for the economic weights; and b is a vector of index weights (coefficients) for the phenotypic values of the selection criteria (Table 5).

**Table 1.** Least-squares means of mature live weight and semen quality parameters of different graded HF × L crossbred bulls of CCBDF.

Trait	Genotype (Least-squares mean ± SE)^1^			
	50%HF × 50%L	62.5%HF × 37.5%L	75%HF × 25%L	Level of sig.
Mature live weight (kg)	659.93ab ± 5.10(8)	670.38a ± 6.97(12)	647.20b ± 4.32(31)	(0.0009)***
Volume of semen (ml)	7.58b ± 0.16(82)	8.82a ± 0.28(134)	8.55a ± 0.18(296)	(0.0041)**
Concentration of semen (106/ ml)	952.80b ± 21.74(85)	1057.85a ± 19.12(135)	1110.67a ± 14.23(296)	(0.0000)***
Mass motility of semen (%)	58.45b ± 0.91(211)	62.69a ± 0.81(297)	62.42a ± 0.54(717)	(0.0003)***
Post-thaw motility (%)	52.55b ± 1.02(209)	55.25ab ± 0.83(299)	55.91a ± 0.56(713)	(0.0100)*

1 Means within a row with no common superscripts differ significantly at * = p < 0.05; ** = p < 0.01 and *** p < 0.001. Values in the parentheses represent the number of observations.

**Table 2.** Descriptive statistics for productive and reproductive traits of daughters of different graded HF × L crossbred bulls.

Trait^1^	Genotype	N	Min	Max	Mean ± SE	CV%	LS^2^
BWT (kg)	HF50% × L50%	959	15	40	24.60^c^ ± 0.14	17.23	(0.0000)***
	HF62.5% × L37.5%	822	15	38	25.61^b^ ± 0.16	18.38	
	HF75% × L25%	2589	15	40	27.20^a^ ± 0.09	17.33	
	HF50% × L50%	958	12	36	22.17^a^ ± 0.15	20.57	
AFC (m)	HF62.5% × L37.5%	828	12	36	20.45^b^ ± 0.13	17.70	(0.0000)***
	HF75%–L25%	2594	12	36	19.66^c^ ± 0.07	18.16	
	HF50%–L50%	954	1	5	1.66^a^ ± 0.02	42.75	
SPC (no)	HF62.5%–L37.5%	825	1	4	1.56^b^ ± 0.02	42.08	(0.0003)***
	HF75%–L25%	2594	1	7	1.60^b^ ± 0.01	44.96	
	HF50%–L50%	954	3	20	6.59^c^ ± 0.07	34.79	
DMY (l/d)	HF62.5%–L37.5%	828	3	16	7.48^b^ ± 0.09	32.69	(0.0000)***
	HF75%–L25%	2594	3	20	8.55^a^ ± 0.06	34.17	
	HF50%–L50%	963	2.5	22	8.05^c^ ± 0.09	33.78	
PMY (l/d)	HF62.5%–L37.5%	828	3	20	9.32^b^ ± 0.11	32.45	(0.0000)***
	HF75%–L25%	2593	3	24	10.44^a^ ± 0.07	32.58	
	HF50%–L50%	928	120	360	215.32^b^ ± 1.65	23.38	
LL (d)	HF62.5%–L37.5%	823	120	330	218.20^b^ ± 1.50	19.70	(0.0000)***
	HF75%–L25%	2563	120	350	233.53^a^ ± 0.85	18.34	
	HF50%–L50%	959	50	240	107.48^a^ ± 1.19	34.41	
DO (d)	HF62.5%–L37.5%	817	50	240	110.19^a^ ± 1.33	34.46	(0.0000)***
	HF75%–L25%	2563	50	240	101.13^b^ ± 0.66	33.18	
	HF50%–L50%	964	10	21	13.09^b^ ± 0.05	12.31	
CI (m)	HF62.5%–L37.5%	826	10	25	13.69^a^ ± 0.08	16.59	(0.0000)***
	HF75%–L25%	2562	10	25	12.97^b^ ± 0.04	13.94	

1 BWT = Birth weight of calf, AFC = Age at first conception, SPC = Service per conception, DMY = Daily milk yield, PMY = Peak milk yield, LL = Lactation length, DO = Days open, and CI = Calving interval.

2 Means within a column with no common superscripts differ significantly at *** = p < 0.001. SE: Standard error, CV: Coefficient of variation, and N= No. of observation.

**Table 3.** Variance components and heritability estimates for productive and reproductive traits of HF×L crossbred daughters of selected breeding bulls.

Trait^1^	σ^2^_*A*_	σ^2^_*E*_	σ^2^_*P*_	h^2^ ± SE
BWT (kg)	3.42 ± 0.95	17.95 ± 0.83	21.37 ± 0.50	0.16 ± 0.04
AFC (m)	3.78 ± 0.81	10.41 ± 0.65	14.19 ± 0.36	0.27 ± 0.05
SPC (no.)	0.05 ± 0.02	0.44 ± 0.02	0.48 ± 0.01	0.09 ± 0.04
DMY (l/d)	1.66 ± 0.32	5.32 ± 0.27	6.99 ± 0.17	0.24 ± 0.04
PMY (l/d)	2.46 ± 0.48	7.05 ± 0.39	9.50 ± 0.23	0.26 ± 0.05
LL (d)	263.29 ± 68.99	1242.60 ± 59.55	1505.90 ± 35.84	0.17 ± 0.04
DO (d)	141.94 ± 38.89	705.53 ± 33.62	847.47 ± 20.05	0.17 ± 0.04
CI (m)	1.07 ± 0.19	2.21 ± 0.15	3.28 ± 0.08	0.32 ± 0.05

1 BWT = Birth weight of calf, AFC = Age at first conception, SPC = Service per conception, DMY = Daily milk yield, PMY = Peak milk yield, LL = Lactation length, DO = Days open, and CI = Calving interval. σ^2^_*A*_ = additive genetic variance, σ^2^_*E*_ = residual variance, σ^2^_*P*_ = total phenotypic variance.A E P

**Table 4.** Estimates of genetic (above diagonal) and phenotypic correlation (below diagonal) among the studied traits in HF × L crossbred daughters of selected breeding bulls.

Trait^1^	1	2	3	4	5	6	7	8
BWT (1)		–0.14 (0.18)	0.33 (0.27)	0.06 (0.17)	0.07 (0.17)	–0.17 (0.20)	–0.12 (0.21)	0.20 (0.16)
AFC (2)	–0.07 (0.02)		0.35 (0.22)	–0.34 (0.14)	–0.40 (0.13)	–0.17 (0.18)	0.17 (0.18)	0.06 (0.14)
SPC (3)	–0.02 (0.01)	0.016 (0.02)		–0.16 (0.23)	–0.16 (0.23)	–0.24 (0.29)	0.19 (0.27)	0.01 (0.21)
DMY (4)	0.23 (0.01)	–0.25 (0.01)	–0.02 (0.02)		0.97 (0.01)	–0.06 (0.17)	0.11 (0.17)	0.20 (0.13)
PMY (5)	0.26 (0.02)	–0.26 (0.01)	–0.03 (0.01)	0.92 (0.00)		–0.06 (0.17)	0.14 (0.17)	0.29 (0.13)
LL (6)	0.06 (0.02)	–0.05 (0.02)	–0.06 (0.01)	0.02 (0.02)	0.08 (0.01)		0.04 (0.20)	–0.08 (0.16)
DO (7)	–0.04 (0.01)	0.12 (0.01)	0.03 (0.01)	0.09 (0.02)	0.06 (0.02)	–0.11 (0.02)		0.50 (0.14)
CI (8)	0.06 (0.02)	0.07 (0.02)	0.01 (0.02)	0.02 (0.01)	0.03 (0.01)	–0.15 (0.01)	0.25 (0.01)	

1 BWT = Birth weight of calf, AFC = Age at first conception, SPC = Service per conception, DMY = Daily milk yield, PMY = Peak milk yield, LL = Lactation length, DO = Days open, and CI = Calving interval. Values in the parentheses represent the standard error of the respective value.

**Table 5.** Weighting factor for the investigated traits.

Trait^1^	BWT	AFC	SPC	DMY	PMY	LL	DO	CI
Economic weight	–8.93	–12.61	–30.91	–20.36	–4.03	–0.98	0.44	29.86

1 BWT = Birth weight of calf, AFC = Age at first conception, SPC = Service per conception, DMY = Daily milk yield, PMY = Peak milk yield, LL = Lactation length, DO = Days open, and CI = Calving interval.

The selection index was constructed using the following formula:

I=b1X1+b2X2+b3X3+…………+bnXn

where b’s are the relative economic value corresponding to each trait and X’s are the standardized breeding value of each trait.

## Results

### Semen quality parameters of breeding bulls

Genotype had significant effects (*p <* 0.05) on the studied semen quality attributes and average live weights of breeding bulls (Table 1). The semen quality traits of 75%HF × 25%L and 62.5%HF × 37.5%L crossbred bulls were significantly better than the 50%HF × 50%L bulls, while non-significant differences were observed between the former two groups of crossbred bulls. The maximum volume of semen was produced by 62.5%HF × 37.5%L crossbred bulls (8.82 ± 0.28 ml) followed by 75%HF × 25%L (8.55 ± 0.18 ml) and 50%HF × 50%L (7.58 ± 0.16 ml) crossbreds. The concentration of semen was higher in 62.5%HF × 37.5%L crossbred bulls (1057.85 ± 19.12 × 106/ml) than in 50%HF × 50%L crossbred bulls (952.80 ± 21.74 × 106/ml). The overall post-thaw motilities of 50%HF × 50%L, 62.5%HF × 37.5%L, and 75%HF × 25%L crossbred bulls were 52.55 ± 1.02, 55.25 ± 0.83, and 55.91 ± 0.56%, respectively.

### Daughters’ productive and reproductive performance

Table 2 describes the descriptive statistics of eight productive and reproductive traits. There were highly significant differences (*p <* 0.01) observed among the genotypes for all considered traits. Daughters of 75%HF × 25%L crossbred bulls showed significantly better performance (*p <* 0.001) for BWT (27.20 ± 0.09 kg), DMY (8.55 ± 0.06 l), PMY (10.44 ± 0.07 l), and LL (233.53 ± 0.85 days). The corresponding values for the daughters of 62.5%HF × 37.5%L and 50%HF × 50%L crossbred bulls were found to be 25.61 ± 0.16 and 24.60 ± 0.14 kg, 7.48 ± 0.09 and 6.59 ± 0.07 l, 9.32 ± 0.11 and 8.05 ± 0.09 l, and 218.20 ± 1.50 and 215.32 ± 1.65 days, respectively. Moreover, 75%HF × 25%L crossbred bulls’ daughters had the lowest AFC (19.66 ± 0.07 months), DO (101.13 ± 0.66 days), and CI (12.97 ± 0.04 months). Daughters of 62.5%HF × 37.5%L crossbred bulls showed longer DO (110.19 ± 1.33 days) and CI (13.69 ± 0.08 months) than the other groups. SPC was found significantly better (*p <* 0.001) in the daughters of 62.5%HF × 37.5%L (1.56 ± 0.02) and 75%HF × 25%L (1.60 ± 0.01) compared to 50%HF × 50%L (1.66 ± 0.02) crossbred bulls.

### Estimation of genetic parameters

The estimates of variance components and heritability are presented in Table 3. The h^2^ estimates of the studied traits were mostly moderate, except SPC. The h^2^ estimates of BWT, DMY, PMY, and LL were 0.16 ± 0.04, 0.24 ± 0.04, 0.26 ± 0.05, and 0.17 ± 0.04, respectively. The reproductive traits were found to be low to moderately heritable traits where h^2^ of AFC, SPC, DO, and CI were determined to be 0.27 ± 0.05, 0.09 ± 0.04, 0.17 ± 0.04, and 0.32 ± 0.05, respectively. Table 4 presents the genetic and phenotypic correlations among the studied traits. The genetic and phenotypic correlations between DMY and PMY were found to be positive and strong, which were 0.97 and 0.92, respectively. DO and CI also had positive but moderate correlations for both genotypic (0.50) and phenotypic (0.25) levels. Genetic correlations of DMY with reproductive traits (AFC, DO, and CI) were found to be heterogeneous and ranged between -0.34 and 0.20, and similar trends were also observed in the phenotypic correlations. SPC had negative genetic correlations (-0.16 to -0.24) with productive traits (DMY, PMY, and LL) but had close to zero to moderate positive correlations with reproductive traits AFC, DO, and CI (0.01–0.35).

### Selection index and ranking of bulls

Among the top 20% of breeding bulls (11 individuals), eight of them were 75%HF × 25%L crossbreds; the remaining 03 sires belong to 62.5%HF × 37.5%L crossbred groups. The selection index was constructed using varying levels of economic weights, which were calculated by a matrix of variances and covariances among the observed phenotypes and genetic covariances between the observed selection criteria and the traits in the objective. Ranking of breeding bulls was performed according to their index values (Table 6). The animals’ actual ID was replaced by numeric IDs in order to maintain trade secrecy. Only 20% of top-ranked animals, their predicted transmitted ability (PTA), and the reliability of each trait are shown in Table 6. The index values of the top three sires were 202.53, 128.09, and 124.47, where the genetic propositions of sires were 75%HF × 25%L. Most of the breeding bulls possessed positive PTA values for productive traits; those ranged between -2.65 and 0.75 for BW, -1.57 and 1.13 for DMY, -1.59 and 1.67 for PMY, and -5.84 and 13.82 for LL, with an accuracy ranging from 0.51 to 0.99. However, the PTA values of the reproductive traits of selected breeding bulls were mostly heterogeneous and varied between -2.32 and 0.772 for AFC, -0.17 and 0.02 for SPC, -9.54 and 7.41 for DO, and -0.55 and 1.67 for CI.

**Table 6.** Rank order of top 20% breeding bulls based on their index values.

Bull ID	Genotype	N^1^	Index Value	Rank order	BW	AFC			SPC	DMY			PMY	LL			DO	CI		
					PTA	AC	PTA	AC	PTA	AC	PTA	AC	PTA	AC	PTA	AC	PTA	AC	PTA	AC
101	75%HF × 25%L	41	202.53	1	–0.25	0.81	–2.32	0.85	–0.04	–	0.26	0.76	0.51	0.81	–5.84	0.98	7.41	0.97	1.67	0.72
102	75%HF × 25%L	74	128.09	2	–0.34	0.85	0.67	0.88	–0.17	–	–0.81	0.80	–1.21	0.85	13.82	0.98	–3.46	0.98	–0.14	0.78
103	75%HF × 25%L	127	124.47	3	0.54	0.87	0.37	0.90	–0.17	–	0.64	0.84	0.91	0.87	3.99	0.99	3.81	0.98	0.88	0.82
104	62.5%HF × 37.5%L	30	106.57	4	–1.39	0.79	–0.79	0.82	–0.08	–	–0.29	0.70	–0.67	0.77	6.78	0.98	3.07	0.97	0.04	0.66
105	75%HF × 25%L	170	104.18	5	–0.14	0.88	–0.41	0.91	–0.03	–	–0.03	0.85	0.17	0.88	2.26	0.99	–0.29	0.98	0.92	0.83
106	62.5%HF × 37.5%L	85	103.32	6	–2.65	0.83	0.72	0.86	–0.13	–	–0.70	0.77	–0.88	0.82	10.67	0.98	–4.47	0.98	–0.55	0.74
107	75%HF × 25%L	213	82.95	7	0.67	0.89	–0.69	0.91	–0.15	–	0.05	0.86	0.30	0.89	–2.60	0.99	–9.54	0.98	–0.08	0.84
108	75%HF × 25%L	7	60.58	8	–0.04	0.74	–0.57	0.78	–0.02	–	–0.35	0.62	–0.09	0.71	–2.09	0.97	4.61	0.96	0.26	0.51
109	75%HF × 25%L	87	52.83	9	0.08	0.86	–0.58	0.89	0.00	–	–1.57	0.81	–1.59	0.85	13.56	0.98	–1.17	0.98	–0.37	0.79
110	62.5%HF × 37.5%L	48	49.05	10	0.75	0.81	–0.23	0.85	0.02	–	1.13	0.75	1.67	0.81	2.27	0.98	0.79	0.97	1.67	0.71
111	75%HF × 25%L	628	36.37	11	0.40	0.91	0.14	0.93	–0.05	–	–1.13	0.89	–1.31	0.91	3.82	0.99	–4.57	0.99	–0.48	0.87

1 No. of progeny records per breeding bull. PTA = predicted transmitted ability, AC = accuracy of the respective PTA value.

## Discussion

Genetic evaluation is a continuous process for the development of any population, and this study aims to evaluate breeding bulls based on their daughters’ performance that could be used for future selection processes. The magnitude of heritability estimates and breeding values of economically important traits can be a significant indicator for the improvement of a population’s performance.

Semen volume ranged from 7.58 ± 0.16 to 8.82 ± 0.28 ml among the crossbred bulls of this study. Similar to the current findings, Rahman et al. [12] reported that the semen volume in HF × L bulls was 7.19 ± 10.19 ml. Hossain et al. [13] reported semen volume for three upgraded populations (L × F, LF_1_ × F, and LF_2_ × F) as 7.4, 12.8, and 9.9 ml, respectively, which partially agrees with this study. However, a relatively lower semen volume was reported by Ahmed et al. [14], varying from 5.80 ± 0.30 to 6.13 ± 0.28 ml. The concentration of spermatozoa per ml semen of HF × L crossbred bulls agrees with the previous studies of Ahmed et al. [14]. They obtained an average semen concentration of HF cross bulls of CCBDF as 1043.5 ± 93.2 × 106/ml. According to Rahman et al. [12] and Akhter et al. [15], semen concentrations of HF × L crossbred were 1257.34 ± 30.46 × 10^6^/ml and 1225 × 10^6^/ml, respectively, which is slightly higher than in our study. Hossain et al. [13] found the mass motility of Friesian and HF × L was 64.0% and 63.7%, which is almost similar to this study. Ahmed et al. [14] reported that the mass motility and post-thaw motility of Friesian cross bulls’ semen were 60.0 ± 2.20 and 49.4 ± 1.00%, respectively, which are slightly lower than this study. However, Akhter et al. [15] and Rahman et al. [12] recorded higher values of mass motility of semen at 66.61% and 66.72 ± 0.99% in L × F × F and Holstein-Friesian × Zebu crossbred bulls, respectively. Both genetic and non-genetic factors potentially influence semen quality attributes [14], and therefore, the genotype of the bull, age, season, frequency of collection, libido, and management might be the attributing factors for the difference between the previous and present studies.

Daughters’ birth weight of 75%HF × 25%L crossbred bulls (27.20 ± 0.09 kg) was significantly higher than that of 62.5%HF × 37.5%L (26.06 ± 0.19 kg) and 50%HF × 50%L (24.87 ± 0.19 kg) crossbred bulls’ daughters. The mean BWT of this study was higher than the reported values of Famous et al. [16] and Rahman et al. [17]. They found the average BWT of Holstein cross calves to be 23.10 ± 1.21 and 19.06 ± 0.28 kg, respectively. Both sire and dam genotypes primarily lead to variations in the BWT of the calf. In addition, the BWT of daughters of different bulls may vary due to differences in the management system, age of the dam, nutrition level of the dam during the pregnancy period, etc.

The effect of the three genetic groups varied significantly (*p* < 0.001) from each other, where the duration of AFC ranged between 590 and 665 days. The results of this study agree with the findings of Sandhu et al. [18], who reported that the average AFC of Holstein-Friesian heifers was 655.10 ± 10.44 days. However, a bit higher AFC (697.51 ± 8.03 days) was reported by Rafique et al. [19] in Holstein-Friesian heifers. The genotype, dam, plane of nutrition, and genotype-environment interaction were major contributing factors for the discrimination between previous and present studies. This study on SPC agrees with the findings of Famous et al. [16] and Bhuiyan et al. [20], who reported that the average service required per conception was 1.61 ± 0.50 and 1.56 ± 0.06, respectively, in Holstein-Local crossbred cattle of Bangladesh. However, there is disagreement with the findings of Hossain et al. [21], who mentioned a higher value (2.39 ± 0.82) in crossbred cattle than in this study. The variations among the studies might be attributed mostly to non-genetic factors, such as proper time of insemination, proficiency of artificial insemination worker, detection of estrous, and variation of genetic makeup within the breed.

There was significant variation observed in the DMY trait among the daughters of the three groups of breeding bulls. The average DMY was recorded as 6.59 ± 0.07, 7.48 ± 0.09, and 8.55 ± 0.06 l in daughters of three different genetic group bulls, which agrees with the findings of Siddiquee et al. [22] and Rahman et al. [17]. This result contradicts the findings of Adhikary et al. [23], who reported that the average DMY of HF×J and HF×L was 16.3 ± 0.64 and 15.6 ± 0.82 kg/day, which is significantly higher than the present study. In PMY, the highest performance was found in daughters of 75%HF × 25%L crossbred bulls (10.44 ± 0.07 l), followed by daughters performance of 62.5%HF × 37.5%L crossbred bulls (9.32 ± 0.11 l) and 50%HF × 50%L crossbred bulls (8.05 ± 0.09 l). Hasan et al. [24] reported that the PMY for 50.0%, 62.5%, and 75.0% HF inheritance was 7.02 ± 0.5, 8.02 ± 0.5, and 9.50 ± 0.5 l, respectively, which is aligned with the present study. However, Famous et al. [16] found higher PMY (14.35 ± 0.52 l/day) than the present findings in Local and Friesian crossbred dairy cows. The genetic makeup of the animals, feeding regimens, management practices, location, and environmental interactions might influence the variations of DMY and PMY traits [16].

Similar to the current studies, Rahman et al. [17] reported that the lactation periods of L × F and L × F × F were 232.2 ± 1.16 and 266.43 ± 1.18 days, respectively. Famous et al. [16] and Adhikary et al. [23] reported higher LL (277, 278, and 277.27 ± 6.16 days) than the present study in HF × L crossbred cattle of different genetic propositions. In general, higher milk production tends to higher LL despite there being variations observed among the individuals of a breed or population. The days open of this study are similar to the trend of Hossain et al. [21] and Famous et al. [16], where DO were 98.7 ± 41.6, 102.9 ± 58.3, and 94.7 ± 33.6 days in 50.0%, 62.5%–68.8%, and 75.0%–87.5% Friesian cross cows. However, the findings of Temesgen et al. [25] reported higher DO (154 days) than the present study. In dairy cattle breeding, the variations could have been caused by farmers’ failure to recognize post-partum heat, differences in season, and mode of insemination. This, in turn, resulted in a longer time between calving and successful fertilization and eventually affected the duration of DO [25]. The CI of our study aligned with the results of Hossain et al. [21] and Siddiquee et al. [22], who reported the duration as 14.17 ± 2.32 months and 403.37 ± 6.27 days, respectively. However, Bhuiyan et al. [20] and Rahman et al. [17] reported higher CI to be 437.23 ± 1.11 and 475.49 ± 9.16 days, respectively, in different graded Holstein cross cows.

In this study, DMY, PMY, and LL showed moderate h^2^ comprising 0.24 ± 0.04, 0.26 ± 0.05, and 0.17 ± 0.04, respectively. Similar trends of heritability estimate for DMY were reported by Toghiani [26] and Getahun et al. [27] in Iranian Holstein cattle (0.26) and Ethiopian Holstein Friesian-Borana crossbred dairy cows (0.28). Birhanu et al. [28] reported a higher h^2^ estimate for DMY (0.43 ± 0.04) in Holstein Friesian × Boran crossbred cattle. Thorat et al. [29] and Rekaya et al. [30] found similar h^2^ estimates for the PMY trait in Holstein Friesian × Deoni crossbred cows (0.27 ± 0.17) and in the Spanish Holstein population (0.26), respectively. The h^2^ estimate of LL fluctuated largely and ranged between 0.12 ± 0.04 and 0.50 ± 0.18 in different Holstein-derived crossbred cattle populations [20,27,29]. Further, higher h^2^ estimates were reported by Das et al. [31] and Ali et al. [32] for BWT (0.44 ± 030 and 0.32 ± 0.18, respectively) in different grades of dairy cows under sub-tropical conditions.

Heritability estimates of reproductive traits ranged from lower for SPC (0.09 ± 0.04) to moderate for CI (0.32 ± 0.05). Similar to the present findings, the h^2^ estimate of SPC was 0.09 [31] in HF × Pabna cattle, but a higher estimate (0.16 ± 0.05) was reported by Bhuiyan et al. [20] in HF×L crossbred cattle of Bangladesh. Previous studies reported DO and CI as low heritable traits that varied between 0.023 ± 0.006 and 0.14 ± 0.21 [26,32]. In contrast, Bhuiyan et al. [20] found CI as a moderately heritable trait (0.25 ± 0.45) in HF × L crossbred cattle and supported the present findings. Non-genetic factors generally contribute major roles to the expression of reproductive traits. However, moderate heritability estimates for AFC, DO, and CI in this study signify the potential genetic effect. Therefore, the selection response for these traits would be positive for their genetic improvement. Furthermore, moderate heritability estimates for all the considered productive traits reveal that genetic progress can be achieved through the selection of superior breeding animals.

The relative response of any trait in a change of other associated trait or traits is important for the improvement of any particular trait of interest in the breeding program [33]. Ayalew et al. [34] reported that the genetic correlations among production traits (LL, LMY, and 305-d MY) were high (> 0.70) in Ethiopian Holstein cattle and partially agreed with the present study. The genetic correlation of DMY with other productive and reproductive traits ranged from moderately negative −0.34 AFC to strongly positive 0.97 PMY. In addition, positive but low genetic correlations were noticed between DMY and reproductive traits DO and CI ranging from 0.11 to 0.20 and are aligned with the previous study of Worku et al. [35]. The phenotypic correlations between production and reproduction traits showed similar trends where most of the traits were correlated within a range of moderately negative (−0.26) to positive (0.26), except DMY with PMY (0.92), and supported by the findings of Das et al. [36]. The weak genetic correlation among traits indicates the influence of one trait over other traits is minimal in the studied HF × L crossbred population, and if one trait gets improved, another trait will not be affected negatively. Therefore, it is possible to develop a trait independently without deteriorating other traits within the population.

A multi-trait selection index considering economic weights has been adopted in dairy and beef cattle breeding [8,37–39]. The construction of a selection index using best linear unbiased prediction estimates breeding values is the widely used method in livestock breeders [37]. Similar to the present study, selection indices were evaluated for pasture-based production of Nellore cattle considering nine economically important traits [8]. Likewise, the economic selection index was constructed using five different traits for sire selection decisions in Beefmaster cattle [38], which supports our study. In this study, bulls’ identification numbers were encoded as 101–111 by sequential ranks, which included the top 20% of different HF × L crossbred bulls under index models. Only three bulls of the 62.5%HF × 37.5%L crossbred group obtained their position in the sire ranking (Bull ID: 104, 106, and 110) among the constructed index. Importantly, the rank order of the top threebulls was from 75%HF × 25%L crossbreds’ group. There are benefits to multi-trait selection using the economic weightage over single-trait selection, as many traits have different economic significance to breeders and producers, while single-trait selection may lead to undesirable changes in correlated traits [6,37]. Taken together, examples of multi-trait selection index-based ranking in crossbred cattle are rare and are absent in HF × L crossbred cattle of Bangladesh. This study opens a new avenue for selecting top-ranked crossbred breeding bulls based on their andrological features as well as progeny performance data.

## Conclusion

Genotype had significant effects on all of the considered semen parameters and productive and reproductive traits. Overall, the daughters’ performance was found to be significantly better in 75%HF × 25%L crossbred bulls than in the other two genetic groups. The h^2^ estimates of the studied traits were found to be low to moderate. The genetic correlations among the investigated traits were found to be negative to moderately positive, except for the correlation between DMY and PMY. All the top-ranked sires belong to 75%HF × 25%L and 62.5%HF × 37.5%L crossbred groups. Finally, the evaluated information on bulls’ progeny performances could be utilized in the sire selection process at CCBDF to improve further productivity of progenies.

## List of abbreviations

AP gAFC, Age at first conception; BLUP, Best linear unbiased prediction; BWT, Birth weight of calf; CCBDF, Central Cattle Breeding and Dairy Farm; CI, Calving interval; DMY, Daily milk yield; GLM, Generalized linear model; HF, Holstein Friesian; L, Local; LL, Lactation length; PMY, Peak milk yield; PTA, Predicted transmitting ability; SL, Sahiwal; SPC, Service per conception; h^2^, Heritability; σ^2^_A_, Additive genetic variance, σ^2^_P_, Phenotypic variance; r_g_, Genetic correlation; b, Vector of index weights.
